# The phenotypic variability of PAPA syndrome: evidence from the Eurofever Registry

**DOI:** 10.1186/1546-0096-13-S1-O8

**Published:** 2015-09-28

**Authors:** R Caorsi, D Marotto, A Insalaco, A Marzano, J Frenkel, A Martini, F De Benedetti, M Gattorno

**Affiliations:** 1 G. Gaslini Institute, 2nd division of Pediatrics, Genova, Italy; 2 Tempio Pausania Hospital, Department of Rheumathology, Tempio Pausania, Italy; 3 Ospedale Pediatrico Bambino Gesù, Department of Pediatrics, Roma, Italy; 4 Fondazione Ca’ Granda, Ospedale Maggiore Policlinico, Department of Dermatology, Milano, Italy; 5 University Medical Center, Department of Pediatrics, Utrecth, Italy; 6 Universisty of Genova, department of Pediatrics, Genova, Italy; 7 The Paediatric Rheumatology International Trial Organization (PRINTO) and the Eurofever Project, Genova, Italy

## Introduction

PAPA syndrome is a very rare autoinflammatory condition. Few data are nowadays available about the clinical characteristic, the response to treatment and the outcome of this disease.

## Objective

To analyse the data of the PAPA patients enrolled to the Eurofever registry.

## Methods

The data analysed in the study were extracted from the Eurofever registry, which is hosted in the PRINTO website (http://www.printo.it). The patients were included in the study in the presence of mutations in the PSTPIP1 gene or, in genetically negative patients, in the presence of at least two of the following clinical manifestation: recurrent pyogenic arthritis, pyoderma gangrenousm or skin abscess with negative cultural tests. Demographic data, clinical manifestations and response to treatment were analysed.

## Results

In March 2015 baseline and clinical information were available of 3200 patients from 88 centers in the Eurofever registry. Of the 27 patients classified as PAPA syndrome, 4 were excluded from the study. 23 PAPA patients (M: F =10:13), from 6 different centers, fulfilled the inclusion criteria and were therefore analysed: 10 were of the same family, in 4 cases one parent was affected (2 included in the registry), while in other 6 patients the family history was negative(of these 3 patients were genetically negative, while the other 3 had de novo mutations). The mean age at enrolment was 26,22 years (8 paediatric and 15 adult patients). The mean age at disease onset was 5,7 years (range birth - 18 years). The mean age at diagnosis was 24,5 years (range 1,8 - 57,5), with a mean delay of 18,8 years (range 2 months - 50 years). The mutations found in the PSTPIP1 gene were V344I (1pt), E250K (1 pt), E257G (1 pt), E277D (1 pts), A230T (2 pts), and E250Q (11 pts).

The disease course was recurrent in 13 patients, while the other 10 presented a chronic disease course with periodic recrudesces. 20 patients presented an articular involvement during their disease course, while 15 patients presented clinical manifestations affecting the skin (folliculitis in 11, pyoderma gangrenosum in 4, skin abscess in 9 patients);7 and 2 patients presented only the articular and skin involvement respectively. 2 patients complained with suppurative hidradenitis while 10 out of the 23 patients presented clinical manifestations not typical of PAPA syndrome (psoriasis, osteolytic bone lesions, chronic renal failure, muscular abscesses, anaemia and hepatosplenomegaly). 12 patients were treated with NSAID with poor response while steroids caused a complete or partial control of disease manifestations in 5 and 8 patients respectively. Four patients were treated with methotrexate with partial response. Etanercept was used in one patient with complete response, adalimumab in 4 patients (3 partial and 1 complete responders) and anakinra in 5 patients (2 partial and 3 complete responders).

## Conclusions

The study analyses the largest series of PAPA syndrome patients described so far. The wide clinical heterogeneity and the usual presentation with a single manifestation might be responsible for under-recognition of the syndrome.

